# Monitoring of chromatin organization in live cells by FRIC. Effects of the inner nuclear membrane protein Samp1

**DOI:** 10.1093/nar/gkz123

**Published:** 2019-02-22

**Authors:** Cecilia Bergqvist, Frida Niss, Ricardo A Figueroa, Marie Beckman, Danuta Maksel, Mohammed H Jafferali, Agné Kulyté, Anna-Lena Ström, Einar Hallberg

**Affiliations:** 1 Department of Biochemistry and Biophysics, Stockholm University, Svante Arrhenius väg 16B, SE-106 91 Stockholm, Sweden; 2 Institute of Environmental Medicine, Karolinska Institutet SE-171 77 Sweden; 3 Monash Molecular Crystallisation Facility (MMCF), Monash University, VIC 3800, Australia; 4 Lipid laboratory, Department of Medicine, Karolinska Institutet, SE-141 57 Huddinge, Sweden

## Abstract

In most cells, transcriptionally inactive heterochromatin is preferentially localized in the nuclear periphery and transcriptionally active euchromatin is localized in the nuclear interior. Different cell types display characteristic chromatin distribution patterns, which change dramatically during cell differentiation, proliferation, senescence and different pathological conditions. Chromatin organization has been extensively studied on a cell population level, but there is a need to understand dynamic reorganization of chromatin at the single cell level, especially in live cells. We have developed a novel image analysis tool that we term Fluorescence Ratiometric Imaging of Chromatin (FRIC) to quantitatively monitor dynamic spatiotemporal distribution of euchromatin and total chromatin in live cells. A vector (pTandemH) assures stoichiometrically constant expression of the histone variants Histone 3.3 and Histone 2B, fused to EGFP and mCherry, respectively. Quantitative ratiometric (H3.3/H2B) imaging displayed a concentrated distribution of heterochromatin in the periphery of U2OS cell nuclei. As proof of concept, peripheral heterochromatin responded to experimental manipulation of histone acetylation. We also found that peripheral heterochromatin depended on the levels of the inner nuclear membrane protein Samp1, suggesting an important role in promoting peripheral heterochromatin. Taken together, FRIC is a powerful and robust new tool to study dynamic chromatin redistribution in live cells.

## INTRODUCTION

Heterochromatin (densely packed, transcriptionally inactive chromatin) tends to concentrate in the nuclear periphery and around nucleoli, while euchromatin (loosely packed, transcriptionally active chromatin) is mainly found in the nuclear interior and at nuclear pore complexes (1,2). Interphase chromosomes occupy different territories. Gene-poor chromosome regions are spatially separated from gene-rich regions (3) where gene-poor chromosomal regions are mostly located at the nuclear periphery while the gene-rich regions tend to locate in the interior (4,5). Chromosome positioning can also be highly tissue-specific; e.g. chromosome 5 tends to localize to the interior in liver cells but at the nuclear periphery in lung cells (6). The nuclear envelope (NE), surrounding the chromatin, consists of two concentric nuclear membranes, the nuclear pores and the nuclear lamina (7). The inner nuclear membrane (INM) harbors a variety of different transmembrane proteins displaying a great diversity in terms of tissue expression pattern (8). Located directly under the INM is the nuclear lamina, which forms a complex meshwork of intermediate filament proteins called lamins (1,9). Together with INM proteins the nuclear lamins tether the genomic material to the nuclear periphery, usually in a transcriptionally silencing manner (10) by binding to so called lamina-associated domains of chromatin (11). The association of chromatin to the NE is believed to be dynamic and vary between cell types because the majority of the NE proteins are highly tissue-specific. Only ≈15% of the nuclear envelope transmembrane proteins (NETs) identified are shared between muscle, liver and blood (8). NETs and the nuclear lamina accumulate transcription factors and regulators at the nuclear periphery affecting chromatin organization and gene regulation.

Tethering chromatin to the nuclear periphery is one way to organize the genomic material in the nucleus. Chromatin can also be directly modified. There are different histone variants that localize differently in chromatin. The histone variant H3.3 is preferentially incorporated into euchromatin and can be incorporated by replacing H3 independently of replication (12–14). Chromatin is also regulated by a variety of post-translational modifications, i.e., acetylations, methylations and phosphorylations. Acetylation of histones is mostly associated with euchromatin while methylation of histones is more complex. Methylation of Lysine 4 on Histone 3 (H3K4me_2_/me_3_) is associated with transcriptionally active chromatin, while methylation of Lysine 9 (H3K9me_2_/me_3_) marks silent promoters and constitutive heterochromatin (15,16). Together these variants and modifications regulate gene expression and chromatin compaction.

Chromatin organization is intensively studied using techniques such as DamID (17), FISH (18), ChIP (19) or HiC (3). These techniques have been developed for different purposes and have different advantages and limitations in terms of capacity and precision. Immunofluorescence using antibody markers suffers from limited access in compact heterochromatic structures (20). Thus, there is a need for an easy to use method that can monitor the dynamic global chromatin organization in live cells. For this, we developed a novel analysis tool called Fluorescence Ratiometric Imaging of Chromatin (FRIC). FRIC is based on a newly designed tandem vector (pTandemH) expressing H3.3-EGFP (marker for euchromatin) and H2B-mCherry (marker for general chromatin) at stoichiometrically constant levels. Confocal fluorescence microscopy and quantitative image analysis was performed to monitor chromatin redistribution in live cells. We show that FRIC accurately displays epigenetic chromatin reorganization in live cells treated with agents known to affect chromatin organization. Using FRIC, we also show that the INM protein Samp1 promotes heterochromatin distribution in the nuclear periphery of U2OS cells.

## MATERIALS AND METHODS

### DNA constructs

The plasmid containing the H3.3 coding sequence (pBOS-H3.3-HA) was a kind gift from Didier Trouche (21). Expression vectors containing coding sequences of H2B and EGFP (enhanced green fluorescent protein) (pBOS-H2BEGFP-N1) were kind gifts from Hiroshi Kimura (22). pRSET-B containing mCherry coding sequence was received from Nora Ausmees (Department of Biology, Lunds University, Sweden) in agreement with Roger Y. Tsien (Howard Hughes Medical Institute Laboratories at the University of California, San Diego, USA). mCherry was amplified using pRSET-B as a template and primers that introduced BamHI and NotI restriction sites (mCherry-BamHI 5′- agggatccaccggtcgccaccatggtgagcaa and Cherry-NotI 5′- tatgcggccgcttacttgtacagctcgtcctcc). The HA-tag in pBos-H3.3-HA was replaced with a BamHI-NotI fragment of mCherry creating the plasmid pBOS-H3.3-mCherry.

**Figure 1.**
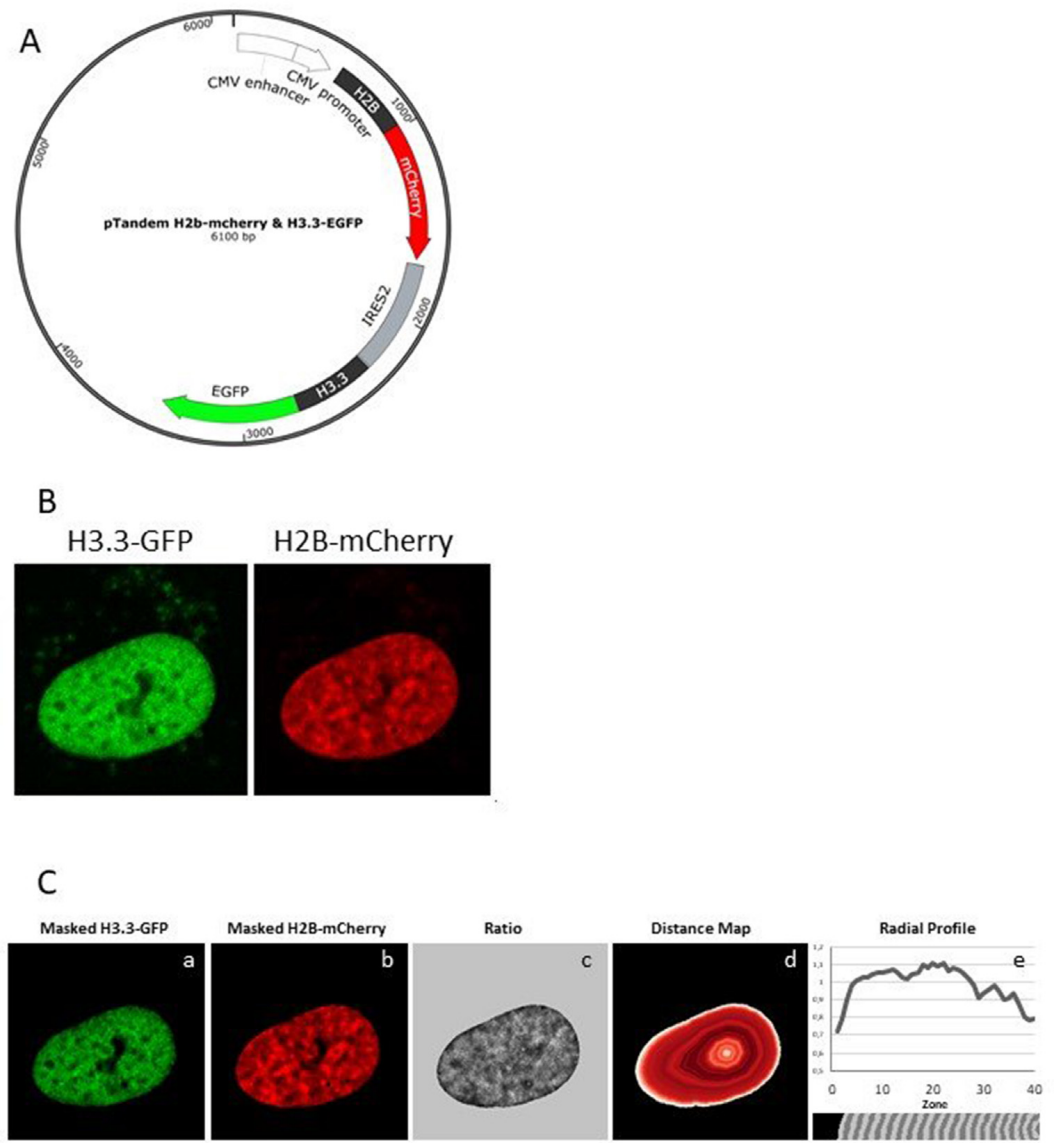
A novel image analysis tool to monitor epigenetic changes in spatiotemporal distribution of chromatin in live cells. (**A**) Schematic illustration of the bicistronic tandem vector (pTandemH) for stoichiometrically constant expression of H2B-mCherry and H3.3-EGFP. (**B**) Confocal fluorescence microscopy images of H3.3-EGFP (left) and H2B-mCherry (right) in live U2OS cells transfected with pTandemH. (**C**) The segmented images from Figure 1B, of H2B-mCherry (a) and H3.3-EGFP (b). The normalized intensity ratio (H3.3/H2B) (c, bright = high ratio, dark = low ratio) plotted on the nuclear distance map as mean ratios (d). Radial profile of zones from the nuclear periphery to the interior (P→I) with incision showing the division of zones in relation to the profile above (e).

The pTandem-1 Vector (Novagen, 71283-3) was designed for co-expression of two genes from a bicistronic messenger RNA. The vector contains separate multiple cloning sites (MCS) on both sides of an internal ribosome entry site, allowing convenient insertion of two open reading frames. H2B-mCherry was polymerase chain reaction (PCR) amplified with a proofreading polymerase to generate blunt ends. The PCR product was then digested with Nco I restriction enzyme and subcloned into the pTandem-1 MCS1 via a blunt end Nco I insertion. H3.3-EGFP was PCR amplified with PCR primers incorporating PmeI and HpaI restriction sites at 5′ and 3′ respectively. The PCR fragment was digested with PmeI and HpaI restriction enzyme and subcloned into the pTandem-1 MCS2 via a blunt end PmeI and HpaI insertion, resulting in pTandemH. Illustration of pTandemH (Figure 1A) was made using the SnapGene software (from GSL Biotech; available at snapgene.com).

For expression of progerin, we used a plasmid encoding Lamin A-L647R prelamin A, which is farnesylated, but cannot undergo endoproteolysis (23). For Samp1 overexpression (Samp1-OX) cells were transfected using a vector termed h.Samp1 (24). As judged by immunofluorescence, overexpressing cells displayed up to 4-fold the endogenous intensity (Figure 6C).

### Cell culture

Human U2OS cells (osteosarcoma) were obtained from ATCC (CCL-2) and cultured in Dulbecco’s modified Eagle’s medium media (Gibco™, 41965-062) supplemented with 10% fetal bovine serum (Gibco™, 10270-106), 1% penicillin-streptomycin (v/v)(Gibco™, 10378-016). The cells were maintained at 37°C in a humidified atmosphere containing 5% CO_2_ and seeded (1–5 × 10^6^ cells) on 35 mm glass bottom dishes (MatTek Corporation, P35G-1.5-20-C) prior to transfection and imaging.

### Transfection

U2OS cells were transfected using X-tremeGENE™ HP DNA Transfection Reagent or XtremeGENE™ 9 Transfection reagent (Sigma-Aldrich) according to the manufacturer’s instructions in a 1:1 or 3:1 (DNA:Reagent) ratio with 1 μg plasmid to 1 or 3 μl X-tremeGENE, incubated for 30 min. The cells were imaged 48–96 h post-transfection.

### Fluorescence microscopy analysis

Images were captured using the confocal laser scanning microscope LSM780 (Carl Zeizz, Germany) using the software Zen Black 2011. Image acquisition was performed using diffraction-limited parameters using Nyquist sampling theorem (z-sections of an interval of 0.551 μm was performed) with pixel size corresponding to 0.0891 um. LD C-Apochromat 63×/1.15 water immersion objective and 63×/1.4 oil immersion objective was used with the 488 nm laser (filter 493–570 nm) and the 561 nm laser (filter 579–632 nm). Cells were kept at 37°C in a humidified atmosphere containing 5% CO_2_ during imaging.

Immunofluorescence of Samp1-KO and Samp1-KD cells were verified Samp1 negative using a 63 × 1.4 NA oil immersion objective and Leica DM/IRBE 2 epi-fluorescence microscope (Leica, Germany). Image intensities were linearly optimized to adjust brightness and contrast to equal intensities using ImageJ (25).

### Imaging program in CellProfiler

Images were segmented using an automatic thresholding strategy with a Gaussian smoothing filter of 5 and by selecting objects above 50 pixels in diameter not touching the image border. Images were then masked from the background and the image quality was measured. Area of the object and intensities were calculated. Images were then normalized by dividing each channel by their mean intensities and variation of each segmented nuclei. A ratiometric image was created dividing the normalized H3.3-EGFP channel by the normalized H2B-mCherry channel. The nucleus was then divided into 40 concentric zones of equal width by dividing the radius into 40 equal parts. The intensity distributions of the ratio were plotted as mean values for each zone, as a radial profile. Finally, the results were saved and exported for further analysis in Excel and GraphPad Prism 7.

Variance was calculated over the inverse ratio images (H2B/H3.3) and compared in the periphery (10 outermost zones) and the entire nuclei of confluent and proliferating U2OS cells, as well as in cells treated with Trichostatin A (TSA), Anacardic acid (AA) or cells overexpressing Progerin or Samp1. A co-occurrence matrix of 8 × 8 pixels was used to calculate the variance as a measurement of structure.

Image Quality was ensured by checking for outliers by the parameters: PowerLogLogSlope (blurriness), PercentMinimal and PercentMaximal (saturation for the object) and the Otsu-threshold (signal-to-noise) for the entire image. Outliers deviating more than 2 SD from average PowerLogLogSlope or with values above 0.2% saturation or 0.15 above Otsu-threshold were manually removed from the dataset. Images were also checked manually for abnormalities.

### dsbCRISPR-Cas9 mediated generation of monoclonal Samp1-KO cell line

The oligos were phosphorylated, annealed and ligated to the pSpCas9(BB)-2A-Puro (PX459) V2.0 vector (Addgene) according to previously described methods (26). Constructs were sequenced for confirmation. Target sequence sgRNA1 (gctgctggcccgctgcccca) was chosen using the E-CRISPR online application (German Cancer Research Center (DKFZ), 2016) for having the highest score and for targeting exon1 in the Samp1 gene. One scrambled sequence with low homology with the human genome was chosen as control.

U2OS cells were transfected with plasmids encoding PAC (puromycin resistance) together with scrambled single guide (sg) RNA and sgRNA1. After selection with 2–10 ug/ml puromycin for 3 days, U2OS colonies were manually transferred to separate 35 mm dishes and cultured. Monoclonal colonies were verified Samp1 negative using both immunofluorescence and western blot analysis.

### Treatments

U2OS cells were transfected with pTandemH, encoding H3.3-EGFP (H3.3) and H2B-mCherry (H2B), for 48–72 h. These U2OS cells were then treated with with 10 μM AA in ethanol (1%, final conc) for 1 h or 50 nM TSA for 24 h or transfected with R-plasmid encoding Progerin, for 48 h. U2OS cells used for Samp1-OX experiments were transfected with pTandem for 48 h and Samp1 for 24 h in confluent U2OS cells, or transfected with both pTandem and Samp1 for 48–72 h in proliferating cells. Stable Samp1-KO cells were transfected with pTandem for 48–72 h. TSA and progerin treated cells were imaged live, while cells treated with AA were washed two times in phosphate buffered saline (PBS) and fixed with paraformaldehyde (PFA) 3.7% for 20 min before being washed three times in phosphate buffered saline (PBS). Control cells for the AA experiment were incubated with 1% ethanol for 1 h. All cells were cultured in 35 mm glass bottom dishes (P35G-1.5-20-C) from MatTek Corporation.

### siRNA treatment

For siRNA-mediated post-transcriptional silencing of Samp1, the following sense sequences were used: Samp1 #1, 5′-GGAAGUGUUGACAGUGUGAtt-3′ (siRNA1) and Samp1 #2, 5′-GCGGCUGUGGAGUACUACAtt-3′ (siRNA2) (27). As scrambled control, Stealth™ RNAi negative universal control (Invitrogen) was used. A total of 18 nM (final conc.) siRNA and 15% (final conc.) HiPerfect (Qiagen) transfection reagent in serum-free medium was used to transfect the cells (day 0 and day 2, after pTandem transfection), which were analyzed after incubation on day 4.

### Immunostaining

All solutions were based on PBS (1×) (Gibco 18912-014). Cells were cultured on no. 1.5 thickness coverslips for the RNApolymerase II, siRNA, Samp1-KO and H3K9me_3_ immunofluorescence experiments. For the former three, coverslips were washed three times for 2 min in PBS on ice, fixed for 20 min in 3.7% paraformaldehyde and permeabilized in 0.5% Triton X-100 for 8 min on ice. This was followed by three PBS washes and blocking in 2% bovine serum albumin containing 0.01% NaN3 (blocking solution) overnight at room temperature. The samples were then incubated overnight with primary rabbit anti-Samp1 antibody (1:500) (27) or mouse anti-RNA polymerase II (1:50) from Abcam (ab24759) in blocking solution, followed by four 2 min washes in blocking solution. The samples were then incubated with secondary anti-rabbit Alexa fluor 488 (1:5000) or anti-mouse Alexa flour 647 (1:5000) from Invitrogen, for 1 h in blocking solution followed by four washes in 0.1% Tween for 2 min each before mounting using Flouromount-G (Southern Biotech) on glass slides, and sealing with nail-polish before imaging. Samples for SIM were treated the same way except cells were cultured on 1.5H coverslips and secondary anti-rabbit Alexa flour 647 (1:5000) from Invitrogen was used. Mounting was done using ProLong™ Gold Antifade Mountant from ThermoFisher. For the H3K9me_3_ staining, the coverslips were washed three times in PBS, followed by 3 × 10 min in 0.1% triton X-100, and blocking in 10% fetal bovine serum for 30 min. Primary rabbit anti-H3K9me_3_ (1:500) from abcam (ab8898) was incubated for 1 h, followed by three washes in 0.1% Triton X-100 and incubation of secondary anti-rabbit Alexa Fluor 647 (1:1000) before mounting as previously stated for RNApolymerase II, siRNA and Samp1-KO.

### Structured illumination microscopy (SIM)

Super-resolution fluorescence 3D-SIM imaging (28) of the labeled cell nucleus was performed on a Zeiss Elyra PS.1. Images were captured with an Andor iXon DU 855 EMCCD camera with a Plan-Apochromate 100×/1.46 NA oil immersion objective. Excitation laser wavelengths used were 488, 561 and 647 nm and fluorescence emission was collected through appropriate dichroic mirrors and single color filters (BP 495–550 nm, BP 570–620 nm and LP 655 nm). An EMCCD-gain of 10–20 and 5 grid rotations were applied with camera integration times of 100–250 ms. Images with 50 nm lateral pixel size were sequentially acquired. Raw SIM datasets were processed with the integrated ELYRA PS.1 system’s analysis software (Zen 2012 SP5 Black) with selection of automatic settings for SIM evaluations (i.e. theoretical PSF, noise filter setting, frequency weighting, baseline handling, etc.). The sectioning (strength) filter settings for the zeroth, first and second orders were 100, 83 and 83, respectively. After processing, rendered SIM images were checked for possible artifacts (e.g. amplified noise patterns) to confirm suitable automatic analysis settings (29). Calibration measurements on 40 nm green fluorescent beads delivering a maximal focal full-width-half-maximum precision of 102 ±5 nm laterally and 250 ±12 nm axially.

### Western blot analysis

Cells were rinsed twice with PBS, scraped into ice cold PBS and centrifuged at 800 × *g* for 5 min. The pellet was resuspended in 1× sodium dodecyl sulphate (SDS) sample buffer, boiled for 5 min and loaded onto 10% sodium dodecyl sulphate-polyacrylamide gel electrophoresis (SDS-PAGE) precast gels (4561034, Bio-Rad). The SDS-PAGE separated proteins were then transferred to a polyvinylidene difluoride (PVDF) membrane (Bio-Rad, 1620177), which was blocked with 5% milk in 0.1% Tween-PBS (blocking solution) for 1 h at room temperature (RT). The membrane was incubated in (1:500) rabbit polyclonal anti-Samp1 antibody in blocking solution for overnight at 4°C. After three 10 min washes in blocking solution, the membranes were incubated in horseradish-peroxidase-coupled donkey antibody (1:5000) against rabbit whole immunoglobulins (IgGs) (GE Healthcare, NA934) in blocking solution for 1 h. After four 10 min washes in PBS containing 0.1% Tween, the membranes were subjected to ECL detection using Amersham ECL Prime western blotting detection reagent (RPN2232, GE Healthcare). The emitted chemiluminescent signal was analyzed by ChemiDoc XRS + imaging system (Bio-Rad). Equal amounts of cell lysate were loaded and Samp1 levels were normalized against β-actin levels.

### Statistical analysis

All datasets were normally distributed, when comparing with their respective bell-curve for zone 1 and zone 2 (data not shown). Graphs and bar charts were presented as mean-values with SEM as error bars. Significance was calculated using unpaired parametric *t*-test (*α* = 0.05) assuming a consistent standard deviation. *P*-values > 0.05 were considered not significant, *P* > 0.01 considered *, *P* > 0.001 **, *P* > 0.0001 *** and *P* < 0.0001 ****.

## RESULTS

### Development of a novel ratiometric image analysis tool for monitoring of epigenetic spatiotemporal distribution of chromatin in live cells

We have developed a novel imaging method called FRIC to be able to measure dynamic chromatin organization in live cells. For this, we have designed a bi-cistronic tandem vector (pTandemH), which ensures stoichiometrically constant expression of fluorescent protein tagged histones (Figure 1A). Histone 3.3 (H3.3) often replaces Histone 3 in transcriptionally active euchromatin, whereas Histone 2B (H2B) is present in all chromatin. Thus, the ratio of fluorescence intensity (H3.3-EGFP/H2B-mCherry) should be a good measure of relative distribution of euchromatin with respect to total chromatin. A high ratio is expected to indicate areas of euchromatin, while a low ratio is expected to indicate areas of heterochromatin. It should be pointed out that FRIC is not designed to register global *de novo* formation or disappearance of euchromatin/heterochromatin, but rather to measure its relative distribution within the nucleus, especially in the nuclear periphery.

To determine the spatial distribution of euchromatin and heterochromatin, the distribution of H2B-mCherry (H2B) and H3.3-EGFP (H3.3) was captured by confocal fluorescence microscopy in optical z-sections in live human osteosarcoma cells (U2OS) transfected with pTandemH (Figure 1B). We have created a quantitative image analysis tool using CellProfiler software (see ‘Materials and Methods’ section), where ratiometric images were created by dividing the EGFP channel with the mCherry channel (i.e. H.3.3/H2B) from the optical section of the nucleus with the largest area, i.e the equatorial section. The nuclei are segmented from the background using an automatic thresholding strategy after size limitations to remove background objects smaller than 50 pixels in diameter (Figure 1C). Images were screened for abnormalities and low quality (signal-to-noise, blurriness and saturation) before being used for measurements (Supplementary Figure S1).

To be able to compare the EGFP and mCherry intensities, the images were preprocessed by normalization of the channels by their specific mean value and variance. The program then divides H3.3 by H2B, creating a ratio image of normalized H3.3/H2B intensities (Figure 1C). A distance-map creating 40 concentric zones of equal width from the nuclear periphery to the interior (P→I) was used to plot a radial profile showing the mean ratio of each consecutive zone (Figure 1C). In an average U2OS cell the nuclear radius is 6 μm, which gives a zone width of ∼150 nm. The example given in figure 1C shows a low ratio (H3.3/H2B) in the first peripheral zones, indicating relatively low euchromatin (high heterochromatin) content in the nuclear periphery, as expected. The zones in the nuclear interior contain less pixels as a consequence of the distance-map and are thus less statistically certain. For more details of the program see Supplementary documents for CellProfiler pipeline. Representation of the dataset as mean ratios in three zones of equal areas, as opposed to equal width is shown as supplementary material (Supplementary Figure S2). In this study, we limited our analyses to the equatorial section with the largest surface area. We consider this approach unbiased because neighboring equatorial sections gave similar radial distribution profiles (Supplementary Figure S3). In contrast, grazing sections were much less informative (Supplementary Figure S3), most likely due to the blurring of zones from neighboring slices, which are more diverse in grazing than in equatorial slices, along the *z*-axis. The latter factor is a limitation that may impose difficulties in future development of 3D-FRIC and restricts us to use FRIC in single slices in this paper.

H3.3 localizes to regions that have the potential to be transcribed (12) however, only ∼70% of the promoters containing H3.3 are transcribed (14). Thus, we expect a partial co-localization of H3.3 and RNA polymerase II (RNApol II). We hypothesized that the ratio (H3.3/H2B) would have a better correspondence with RNApol II than H3.3, as regions containing more chromatin could potentially have higher amounts of H3.3 by chance. The ratio (H3.3/H2B) would instead indicate regions with higher relative amounts of euchromatin. We also assumed that the H3K9me3 heterochromatin marker would correlate poorly with both the ratio and H3.3, but correlate well with H2B and the inverse ratio (H2B/H3.3). To evaluate the correlation between the ratio and RNApol II or H3K9me3 distribution, we chose to utilize both the Pearson Correlation Coefficient (PCC) and the Mander’s Correlation Coefficient (MCC). PCC compares signal intensity through a pixel-by-pixel comparison that compares the deviation from the mean between two channels, while MCC compares the distribution of pixels with an intensity reaching a certain threshold for two channels (30). Cells were transfected with pTandemH and immunostained using antibodies specific for RNApol II or H3K9me3 (Supplementary Figure S4A). RNApol II immunostaining showed a higher PCC and MCC for the ratio compared to H3.3, suggesting that the ratio correlates better with RNApol II than H3.3 alone, while the correlation between the ratio or H3.3 and H3K9me3 was low or negative. The H2B signal, i.e. regions with relatively high amounts of total chromatin and the inverse ration, had a negative PCC and low MCC with RNApol II and a higher correlation with H3K9me3 (Supplementary Figure S4B).

### Non-dividing G_0_ cells display more structured chromatin distribution compared to proliferating cells

**Figure 2.**
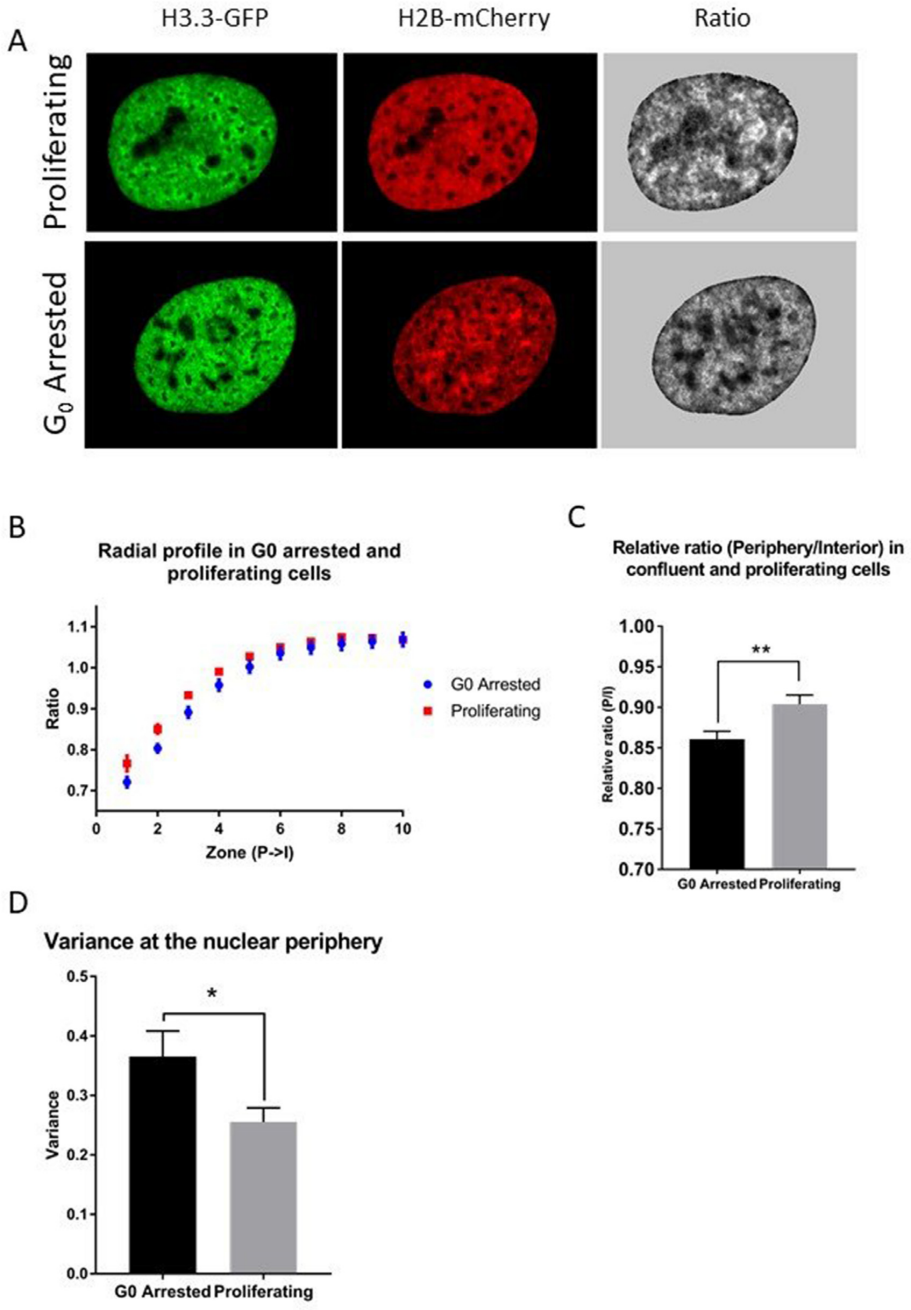
Chromatin distribution in proliferating and G_0_ arrested U2OS cells. (**A**) Fluorescence images of H3.3-EGFP and H2B-mCherry and ratiometric images of proliferating and confluent G_0_ arrested U2OS cells transfected with pTandemH. (**B**) Radial profile of the mean ratio from nuclear periphery to the interior (P→I) of the peripheral zones (zone 1–10, see supplementary for full profile) of proliferating and G_0_ cells. (**C**) Mean relative ratio (P/I, peripheral zone 1–3/ interior zone 4–40) of proliferating and G_0_ cells (*P* = 0.0044). (**D**) The variance of the ratio in proliferating and confluent cells of a zone comprising the 10 most peripheral pixels was higher (*P* = 0.026) in the G_0_ cells (*n* = 90 proliferating, 92 G_0_ cells). Experiments were performed three times. P-values > 0.05 were considered not significant, P > 0.01 considered *, P > 0.001 **, P > 0.0001 *** and P < 0.0001 ****.

It is possible that chromatin distribution varies over the cell cycle. Thus, we tested how this would affect the performance of FRIC by comparing normally proliferating cycling U2OS cells with confluent U2OS cells (Figure 2A), which stop dividing due to contact inhibition and arrest in G_0_ (31). The radial profile of the H3.3/H2B ratio between proliferating and G_0_ U2OS cells was not significantly different (Figure 2B and Supplementary Figure S5). However, when the nuclear periphery (P, zones 1–3) was compared to the interior (I, zones 4–40), the relative ratio (mean ratio (P/I)) displayed significantly less euchromatin (low ratio) in the periphery of confluent G_0_ cells compared to proliferating cells (Figure 2C). Furthermore, in the periphery (defined as the 10 outermost zones) we saw a small increase of variance in confluent G_0_ cells (Figure 2D) suggesting that they have a higher level of structural organization of chromatin in the periphery. When analyzing the entire nucleus however, the variance of the ratio showed no significant change between proliferating and confluent cells. Furthermore, proliferating cells had less variance within individual cells, but had a higher standard deviation between cells (data not shown). This phenomenon can be explained by the fact that the proliferating cells are cycling, whereas confluent cells are arrested in G_0._ The uniformity of G_0_ arrested cells ensures a homogenous test pool of cells, allowing us to observe more minor changes in the distribution of chromatin. Therefore, we chose to use confluent G_0_ U2OS cells to optimize registration of experimentally induced differences in chromatin organization in this study. Future studies on cells synchronized in different ways could potentially demonstrate the usefulness of FRIC for studies of subtle cell cycle variations in chromatin organization.

### The effect of inhibitors affecting histone acetylation on H3.3/H2B distribution

**Figure 3.**
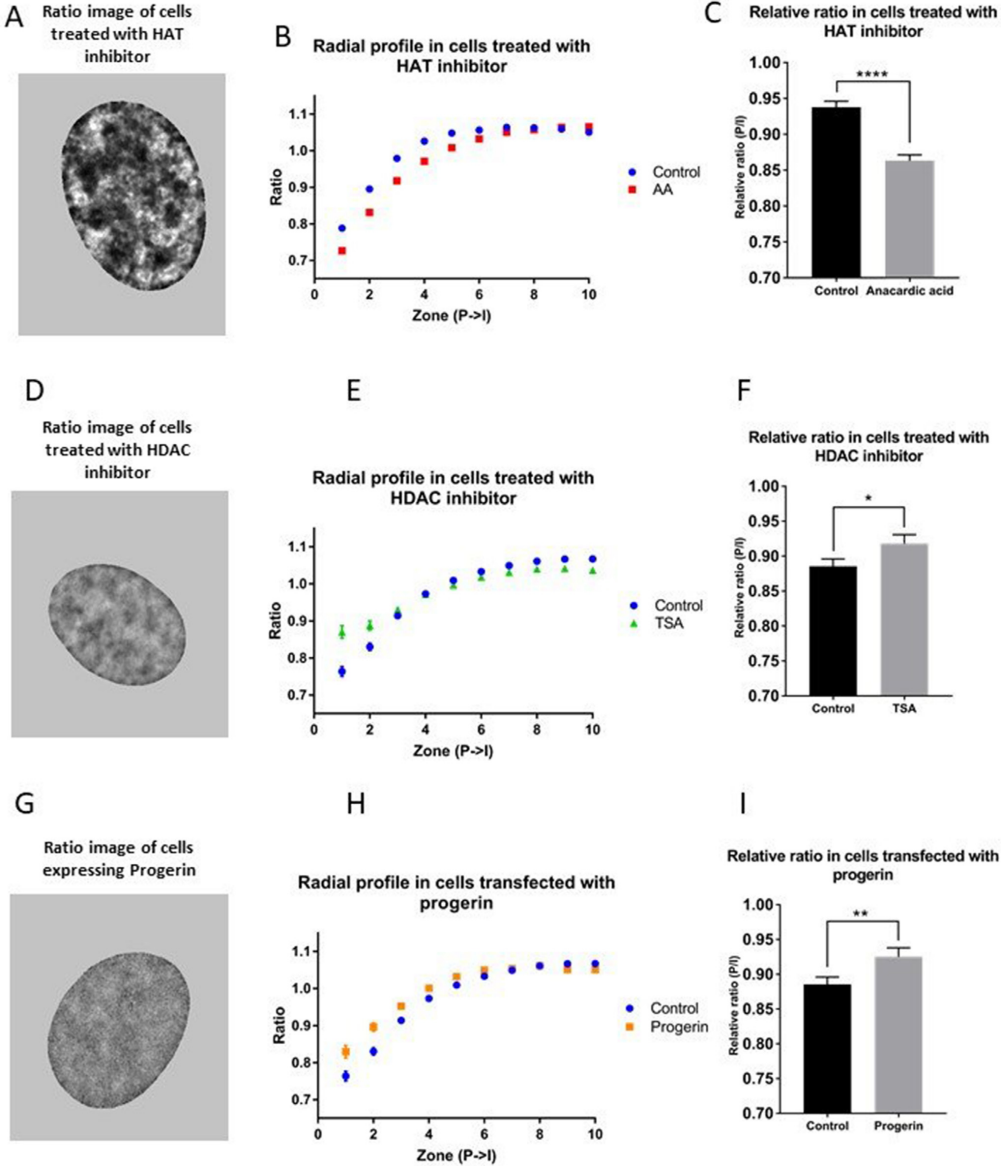
Effects of histone acetylation and Progerin on chromatin organization. U2OS cells transfected with pTandemH were treated with AA (**A**–**C**), TSA (**D**–**F**) or transfected with a plasmid encoding progerin (**G**–**I**). (A) Ratiometric images (H3.3/H2B) of an AA treated U2OS cell. Heterochromatin (darker) and euchromatin (brighter) regions. (B) Radial profile (P→I; only the first 10 zones are shown in the graph; see supplementary for all zones). Cells treated with AA had significantly (*P* < 0.0001) lower ratios in the nuclear periphery (zone 1–5) in comparison to control cells (*n* = 144 AA treated, *n* = 107 control). (C) Mean relative ratio (P/I, zone 1–3/zone 4–40) of AA treated and control cells (*P* < 0.0001). (D) Ratiometric images of TSA treated U2OS cells. (E) Radial profile of cells treated with TSA and control cells (only the first 10 peripheral zones (P→I) are shown in the graph, see supplementary for all zones). Cells treated with TSA had significantly (*P* < 0.00052) higher ratio in the nuclear periphery (zone 1–2) in comparison to control cells (*n* = 45 TSA treated, *n* = 52 control cells). (F) Mean relative ratio (P/I, zone 1–3/zone 4–40) of TSA treated and control cells (*P* = 0.002). (G) Ratiometric images of progerin expressing U2OS cells. (H) Radial profile of cells transfected with progerin and control cells (only the first 10 peripheral zones (P→I) are shown in the graph, see supplementary for all zones). Cells transfected with progerin had significantly (*P* < 0.0103) higher ratio in the nuclear periphery (zone 1–2) in comparison to control cells (*n* = 55 progerin, 52 control cells). (I) Mean relative ratio (P/I, zone 1–3/zone 4–40) of progerin transfected and control cells (*P* = 0.0057). Experiments were performed three times. P-values > 0.05 were considered not significant, P > 0.01 considered *, P > 0.001 **, P > 0.0001 *** and P < 0.0001 ****.

We decided to test the performance of our ratiometric imaging tool by altering chromatin organization experimentally. Inhibition of histone deacetylase (HDAC) activity by TSA is expected to result in loss of heterochromatin (32,33), whereas inhibition of histone acetyl transferase activity by AA is expected to result in loss of euchromatin (34) (35). U2OS cells were transfected with pTandemH and treated either with 50 nM TSA for 24 h or 10 μM AA for 1 h, respectively. Ratiometric images were acquired in confluent cells and the radial distribution (P→I) of the ratio (H3.3/H2B) was calculated. Different treatments and conditions had varied effects in terms of the number of peripheral zones affected. Therefore, we decided to specify the number of zones with statistically significant differences throughout the paper. Cells treated with AA showed a significantly lower ratio in the periphery (zone 1–6), indicating increased heterochromatin in the nuclear periphery (Figure 3A–C, Supplementary Figure S6 for full profile). In contrast, cells treated with TSA showed a significantly higher ratio in the periphery (zone 1–2) in comparison with control cells, indicating increased euchromatin in the nuclear periphery (Figure 3D–F, Supplementary Figure S6 for full profile). In support, TSA treated cells displayed decreased distribution of the heterochromatin marker H3K9me3 in the nuclear periphery (Supplementary Figure S6). Both AA and TSA have known global effects on histone acetylation and FRIC was able to register the counteracting chromatin reorganization in the nuclear periphery induced by these inhibitors.

In order to study the structural changes in the nuclei and especially at the periphery, we analyzed the variance (36) of the inverse ratio (H2B/H3.3), i.e. heterochromatin. The variance was significantly lower in the periphery of TSA treated cells compared to control cells. There was also a significant difference between TSA treated cells and control cells in regard to the entire nucleus, although this was smaller, indicating a decreased level of structural organization of chromatin. In contrast, AA treated cells showed a higher degree of structure in both periphery and in the entire nucleus compared to control cells (Supplementary Figure S7), indicating an increased level of structural organization of chromatin.

### Expression of progerin affects spatial H3.3/H2B distribution

Mutations of different NE proteins are known to change distribution of peripheral chromatin and gene expression. This has been linked to cancer and heritable human diseases, such as laminopathies (37). To test the performance of FRIC for analysis of pathological conditions, U2OS cells were transfected with pTandemH and co-transfected with a plasmid encoding progerin (a pre-lamin A mutant)(23), which causes Progeria in humans and is known to result in loss of peripheral heterochromatin (38). Forty-eight hour post-transfection, most of the U2OS cell nuclei retained an apparently normal morphology but displayed a significantly higher ratio in the periphery, zone 1–2 (Figure 3G–I, Supplementary Figure S6 for full profile), indicating a decrease in heterochromatin in the nuclear periphery. Cells expressing progerin also showed a significant loss of structural organization in the periphery and, to a lower degree, in the entire nucleus (Supplementary Figure S7).

### Monitoring of dynamic chromatin reorganization in cells treated with TSA

**Figure 4.**
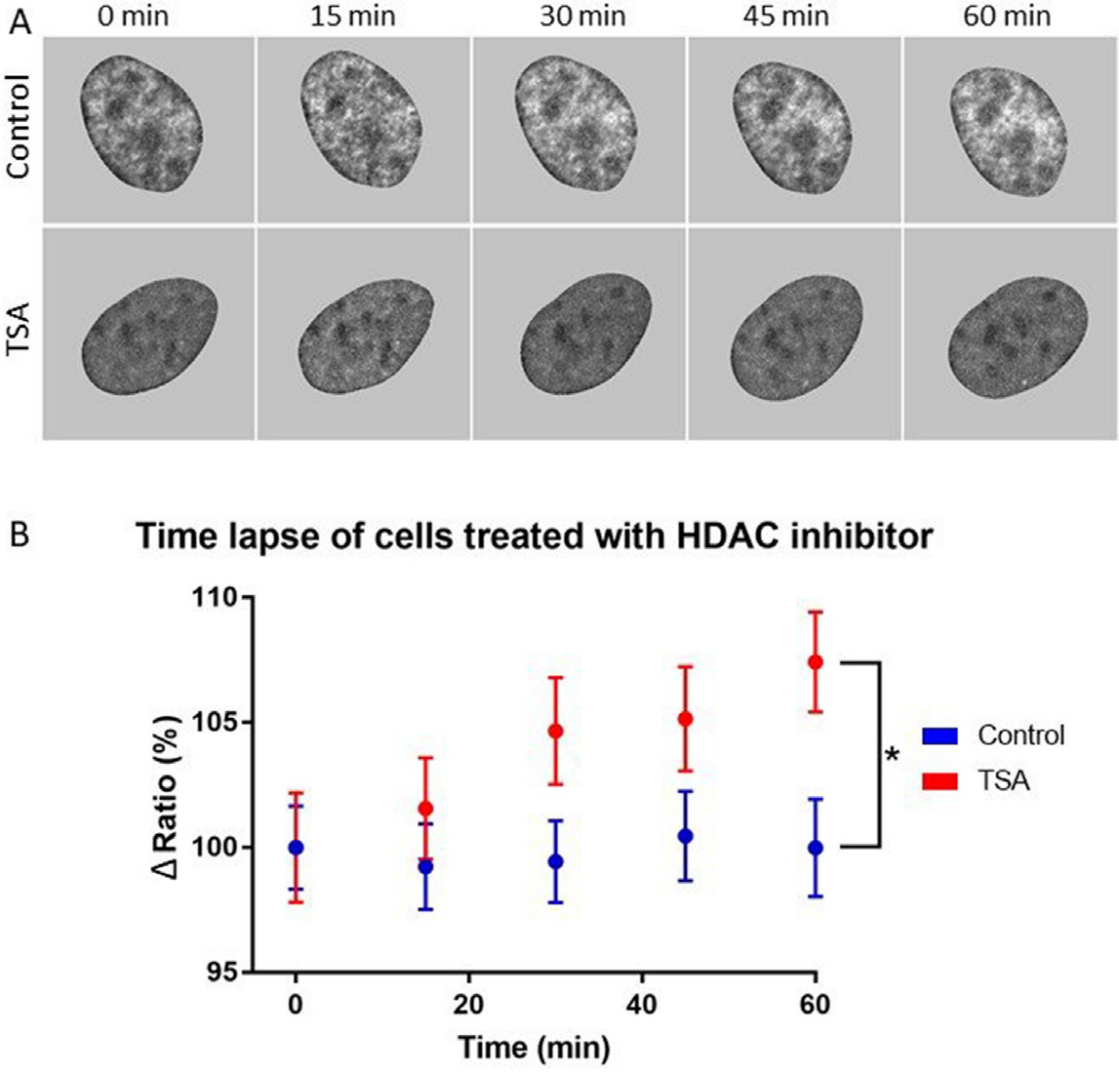
Monitoring of dynamic changes in chromatin organization in live cells treated with TSA. U2OS cells transfected with pTandemH were treated with 200 nM TSA and monitored every 15 min. (**A**) Ratiometric images of control cells and cells treated with TSA after 0, 15, 30, 45 and 60 min. (**B**) Time lapse study displaying change in ratio of the nuclear periphery (zone 1) of control and TSA treated cells. After 60 min, cells treated with TSA had a significantly (*P* = 0.0115) higher ratio in the nuclear periphery (zone 1) in comparison to control cells (*n* = 56 control, *n* = 45 TSA cells). Experiments were performed three times. P-values > 0.05 were considered not significant, P > 0.01 considered *, P > 0.001 **, P > 0.0001 *** and P < 0.0001 ****.

In order to demonstrate that our method is useful in live cells for monitoring and quantitatively analyzing dynamic chromatin reorganization events, we performed time lapse experiments on U2OS cells transfected with pTandemH. The cells were treated with 200 nM TSA at time point 0 and followed for 1 h (Figure 4A). The ratio (H3.3/H2B) in the nuclear periphery (zone 1) increased with time and was significantly higher in cells treated with TSA after 1h (Figure 4B). Despite the short time of exposure to TSA, the effect on the difference in H3.3/H2B ratio in the most peripheral zone was detectable using FRIC.

### The effect of the INM protein Samp1 on chromatin organization

**Figure 5.**
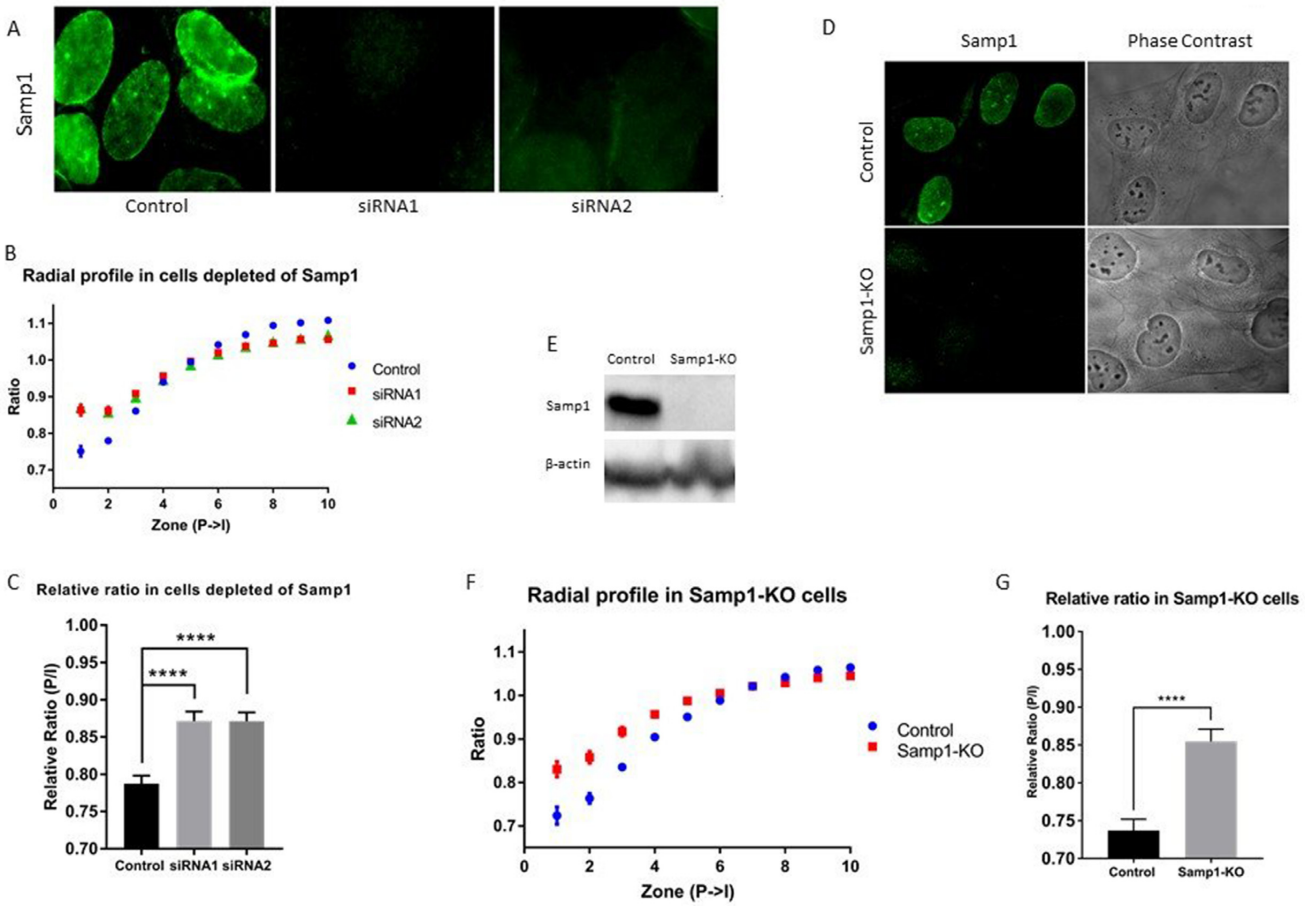
Loss of peripheral heterochromatin in Samp1 depleted cells. U2OS cells were depleted of Samp1 acutely or stably using siRNA and crispr/Cas9 genome editing (**A**) The immunofluorescence signal of the anti-Samp1 staining was dramatically decreased in U2OS cells treated with siRNA1 and siRNA2 against Samp1 (Samp1-KD cells). (**B**) Radial profile of the Samp1-KD (siRNA1 and siRNA2) and control (scrambled) cells (only the first 10 peripheral zones (P→I) are shown in the graph, see supplementary for all zones). The Samp1-KD cells had significantly (*P* < 0.00048) higher ratio in the nuclear periphery (zone 1–3) in comparison to control cells (*n* = 97 siRNA1, 85 siRNA2, 109 scrambled cells). (**C**) Mean relative ratio (P/I, zone 1–3/zone 4–40) of siRNA1 (*P* < 0.0001) and siRNA2 (*P* < 0.0001) in comparison to control cells. (**D**) The Samp1-KO U2OS cells were analyzed for Samp1 expression using immunofluorescence microscopy and (**E**) western blot analysis. Scrambled sgRNA was used as control. (**F**) Radial profile of the Samp1-KO and control (scrambled) cells (only the first 10 peripheral zones (P→I) are shown in the graph, see supplementary for all zones). The Samp1-KO cells had significantly (*P* < 0.00034) higher ratio in the nuclear periphery (zone 1–5) in comparison to control cells (*n* = 66 Samp1-KO, 63 scrambled cells). (**G**) Mean relative ratio (P/I, zone 1–3/zone 4–40) of the Samp1-KO and scrambled cells (*P* < 0.0001). Quantitative experiments were performed three times. P-values > 0.05 were considered not significant, P > 0.01 considered *, P > 0.001 **, P > 0.0001 *** and P < 0.0001 ****.

**Figure 6.**
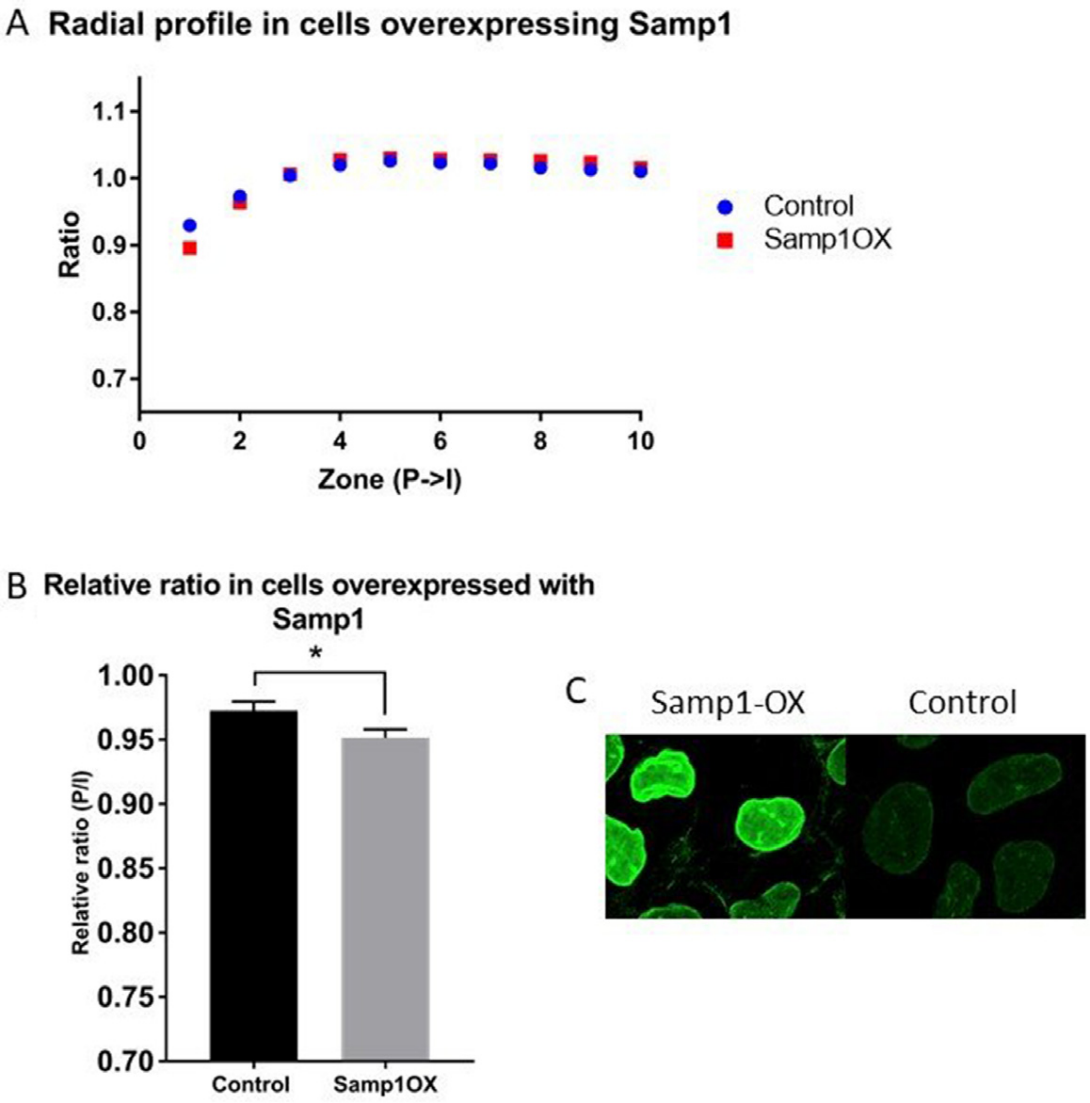
Overexpression of Samp1 increases peripheral heterochromatin. (**A**) Radial profile of U2OS cells transfected with pTandemH and complementary DNA encoding for Samp1 (only the first 10 peripheral zones (P→I) are shown in the graph, see supplementary for all zones). Samp1 overexpressing (Samp1-OX, 4-fold increase) cells had significantly (*P* = 0.00039) higher ratio in the first nuclear peripheral zone 1, in comparison to control cells (*n* = 88 Samp1-OX, 43 control cells). (**B**) Mean relative ratio (P/I, zone 1–3/zone 4–40) of the Samp1-OX cells (*P* = 0.0265) in comparison to control cells. Experiments were performed three times. (**C**) Images showing immunofluorescence intensities of the Samp1-OX cells compared to control cells. P-values > 0.05 were considered not significant, P > 0.01 considered *, P > 0.001 **, P > 0.0001 *** and P < 0.0001 ****.

Proteins in the NE have an important role in regulating chromatin distribution. The INM protein Samp1/Net5 (27) has previously been shown to relocalize chromosome 5 to the nuclear periphery in HT1080 fibroblasts (39) and Ima1, the Samp1 homolog in *Schizosaccharomyces pombe*, attaches centromeric heterochromatin to the nuclear periphery (40). Therefore, we asked the question if altered Samp1 expression could affect chromatin organization in human U2OS cells. First, we acutely knocked down Samp1 using two individual siRNA oligonucleotides (Samp1-KD)(27). The knockdown was confirmed by immunofluorescence microscopy (Figure 5A). Samp1-KD cells displayed a significantly higher ratio in the nuclear periphery (zone 1–3, Figure 5B, Supplementary Figure S8 for full profile), and in the relative ratio (P/I, Figure 5C), suggesting that Samp1 is required for maintaining peripheral heterochromatin. We also wanted to establish if the phenotype lingered after several passages or if the effect could be compensated by other factors over time. To knock out Samp1, U2OS cells were transfected with plasmids encoding Cas9 and sgRNA targeting the Samp1 gene resulting in stable Samp1 knockout (Samp1-KO) U2OS cells. These cells were classified as Samp1 negative using both indirect-immunofluorescence microscopy (Figure 5D) and western blot analysis (Figure 5E). The Samp1-KO cells were transfected with pTandemH and the radial profile was plotted from the nuclear periphery to the interior (Figure 5F, Supplementary Figure S8 for full profile). The Samp1-KO cells showed a significantly higher ratio in the nuclear periphery (zone 1–5) compared to control cells and had a higher relative ratio (P/I) (Figure 5G), suggesting that the loss of Samp1 function in chromatin organization is not compensated by other proteins in U2OS cells. We also performed converse experiments where we increased the levels of Samp1 ∼4-fold by overexpression (Figure 6C). Samp1-OX resulted in a significantly lower ratio in the periphery (zone 1, Figure 6A, Supplementary Figure S8 for full profile), and in the relative ratio (P/I, Figure 6B), corroborating the depletion experiments (Figure 5) and supporting the idea that Samp1 promotes distribution of heterochromatin to the nuclear periphery. Furthermore, cells depleted of Samp1 had a lower structural variation in the nuclear periphery, while Samp1 overexpressing cells displayed more structural variation in the periphery compared to control cells (Supplementary Figure S9).

The higher H3.3/H2B ratio in U2OS cells depleted of Samp1 (KD and KO cells) indicates a reorganization of chromatin, resulting in more euchromatin at the nuclear periphery. The effects of Samp1 depletion or overexpression show that peripheral heterochromatin distribution in U2OS cell nuclei is dependent on Samp1 expression levels and suggest that Samp1 either promotes heterochromatin or prevents the formation of euchromatin in the nuclear periphery.

We then investigated if the same results could be seen using proliferating cells, as compared to confluent G0 cells, to ensure that the method would be sensitive enough for screening cells without the need for synchronization first. Indeed, we were able to replicate the results when comparing proliferating control cells to proliferating Samp1-KO cells or proliferating Samp1-OX cells (Supplementary Figure S5), suggesting that FRIC can be applied to monitor subtle changes in chromatin arrangement also in asynchronous cultures.

### Structured Illumination Microscopy on Samp1-KO cells

**Figure 7.**
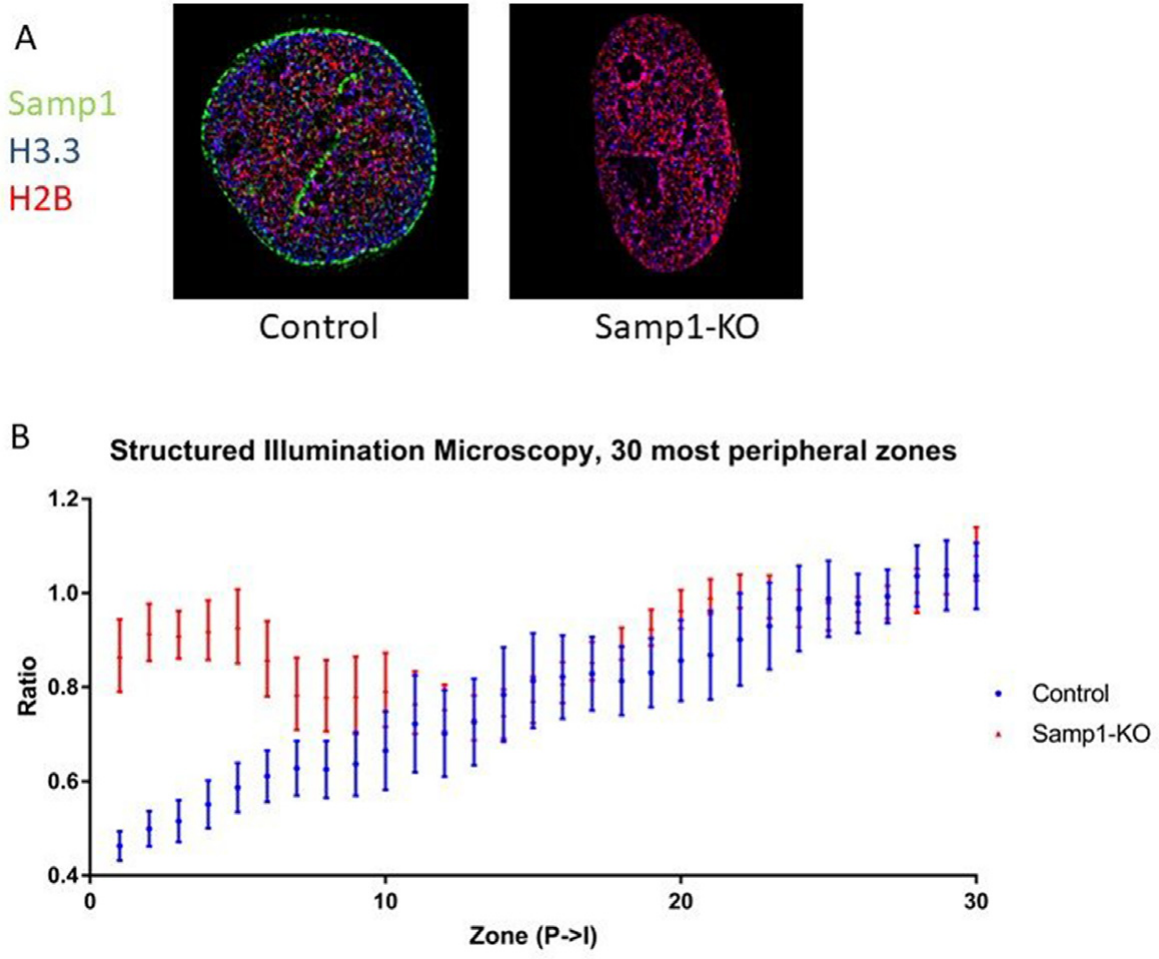
SIM on cells depleted of Samp1. U2OS cells were depleted of Samp1 using crispr/Cas9 genome editing as described in Figure 5D, transfected with pTandemH and immunostained with antibodies specific for Samp1. (**A**) Merged SIM images of nuclei of control and Samp1-KO cells, respectively. (**B**) Mean relative ratio (P→I, 30 most peripheral zones) of control (*n* = 9) and Samp1-KO (*n* = 7) cells, respectively, presented with SEM error bars. The four most peripheral zones were significantly different from controls (*P* < 0.041).

To test if FRIC was compatible with super resolution microscopy, to enable even more sensitive observation of the distribution of chromatin at the NE, we performed Structured Illumination Microscopy (SIM) of Samp1-KO cells and control cells, transfected with pTandemH and immunostained for Samp1 (Figure 7A). The increased resolution of SIM enabled visualization of effects of Samp1 depletion on chromatin organization in at least 10 zones (Figure 7B, Supplementary Figure S8 for full profile). Thus, FRIC is compatible with super resolution and provides a more detailed picture of the chromatin distribution in the nuclear periphery, which is of great interest when studying the role of NETs in chromatin organization.

## DISCUSSION

Here we present FRIC, as a new straight forward, easy and versatile method to visualize and quantify spatial epigenetic chromatin reorganization events in single live cells. The method is robust, as the pTandemH vector assures a stoichiometrically constant relation between the two fluorescent fusion proteins. Both H3.3 and H2B have previously been expressed as fluorescent protein fusion proteins under the CMV promoter with no signs of abnormality or toxicity (14,41). The ectopic expression of the fusion proteins is expected to contribute little to the vast excess of endogenous histone molecules and therefore has an estimated minimal 2% effect on cellular transcription rates (14). FRIC is especially useful for studies of dynamic global systemic chromatin reorganization events and is a valuable asset in investigations of mechanisms behind cell differentiation and development of pathological conditions, such as laminopathies or cancer and could potentially be used in diagnostic investigations of patient cell biopsies. Additionally, FRIC can be used to study global chromatin rearrangement during different stages of the cell cycle on a single cell scale, providing valuable information about arrangement of chromatin near the NE.

The method can be combined with the use of third color reporters for visualization of specific loci or nuclear sub-compartments. Since FRIC is based on fluorescent protein-tagged histones it does not suffer from accessibility problems associated with immunostaining (20). The pTandemH vector is versatile and can be used to transfect any mammalian cell type, a feature that may be especially helpful to understand the mechanism(s) behind laminopathies, which affect many different tissues. The ability to investigate chromatin organization events in single live cells can be advantageous in comparison to cell population based techniques, which suffer from problems in detecting rare transient events and to distinguish frequent transient events from infrequent stable situations. FRIC is also unbiased in terms of cross linker or antibody accessibility which could be problematic in dense chromatin regions.

We also show that FRIC is compatible with super resolution imaging (Figure 7), which significantly increases possibilities to perform detailed studies of the role of NE proteins in chromatin tethering to the NE. At least two structures involving transmembrane INM proteins, the lamin B-dependent ‘B-tether’ and the lamin A-dependent ‘A-tether’, contribute to distribution of interphase chromatin in the nuclear periphery (42). In the present study, we found that peripheral chromatin distribution depended on Samp1 expression levels (Figure 5 and 6), suggesting that Samp1 promotes organization heterochromatin in the periphery of U2OS cell nuclei. We cannot at this stage conclude if this is due to involvement in tethering or some other type of remodeling event. In a previous attempt to identify proteins involved in the A-tether, which is disrupted in rod cells of nocturnal (but not in diurnal) animals, expression of Samp1 did not correlate with a functioning A-tether (43). Given the large diversity in expression of INM proteins (8), tethering mechanisms can vary between cell types (44). The effect of Samp1 in U2OS cells can be either direct or indirect. In all cell types investigated so far, Samp1 binds emerin (24,45,46), which in turn binds to the chromatin protein barrier-to-autointergration factor (BAF) (47). In U2OS cells Samp1 interacted with lamin B (46), a component of the B-tether and could thus interact with this network. Future investigations will be needed to unravel the mechanism of Samp1 in organizing chromatin in the nuclear periphery.

## Supplementary Material

Supplementary Data
