# Supplementary material for: Monitoring of chromatin organization in live cells by FRIC. Effects of the inner nuclear membrane protein Samp1
Source: Nucleic Acids Res. 2019 Feb 22;47(9):e49. doi: 10.1093/nar/gkz123 (PMC6511872; doi:10.1093/nar/gkz123)
Supplement: Supplementary Data [file gkz123_supplemental_files.zip › Supplementary Images, submission 4 20190117.pdf]

Supplementary Figure S1

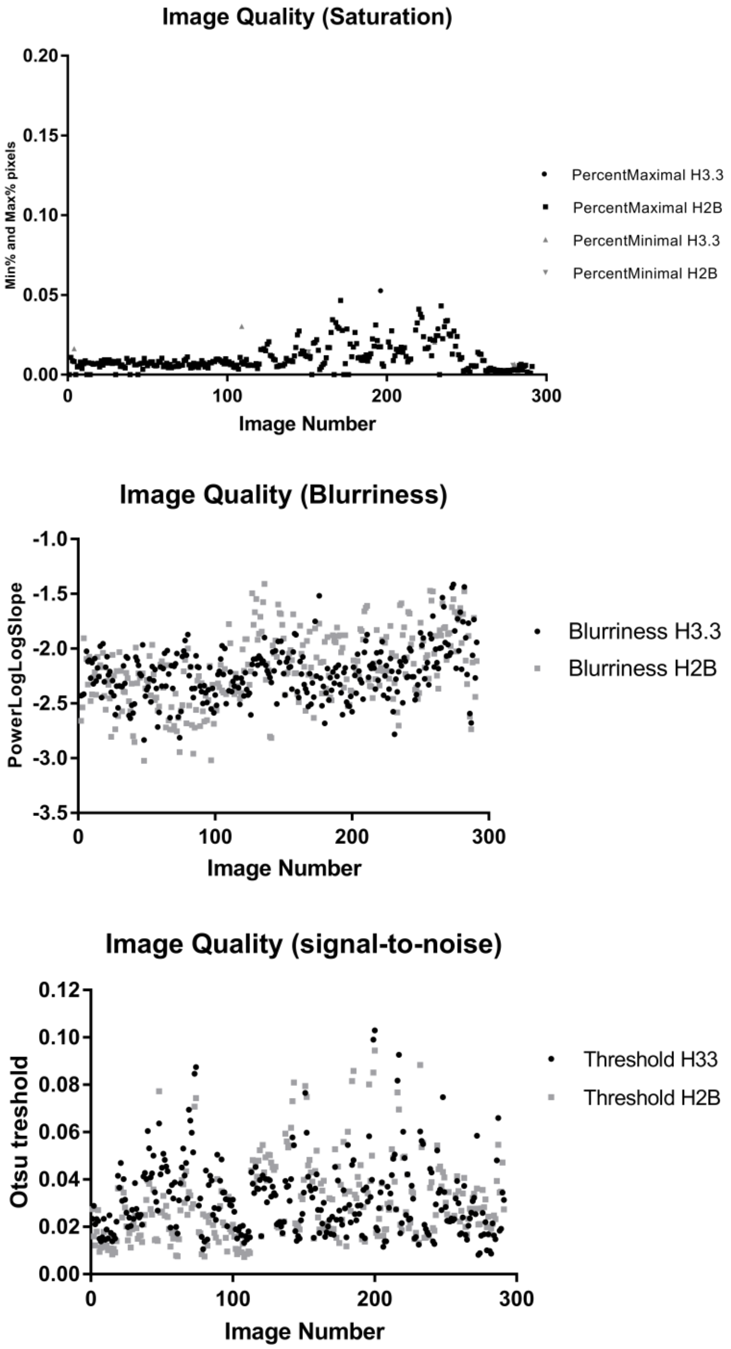

Supplementary Figure S1. Example of image quality assessment of cells in Confluent vs Proliferating, TSA, Progerin, Samp1-KO and Samp1-KD experiments. Images with acceptable image quality in regard to saturation (lower than 0.2%), blurriness (within 2 standard deviations from average PowerLogLogSlope), and signal-to-noise ratio (lower than 0.15 above Otsu-threshold).

Supplementary Figure S2

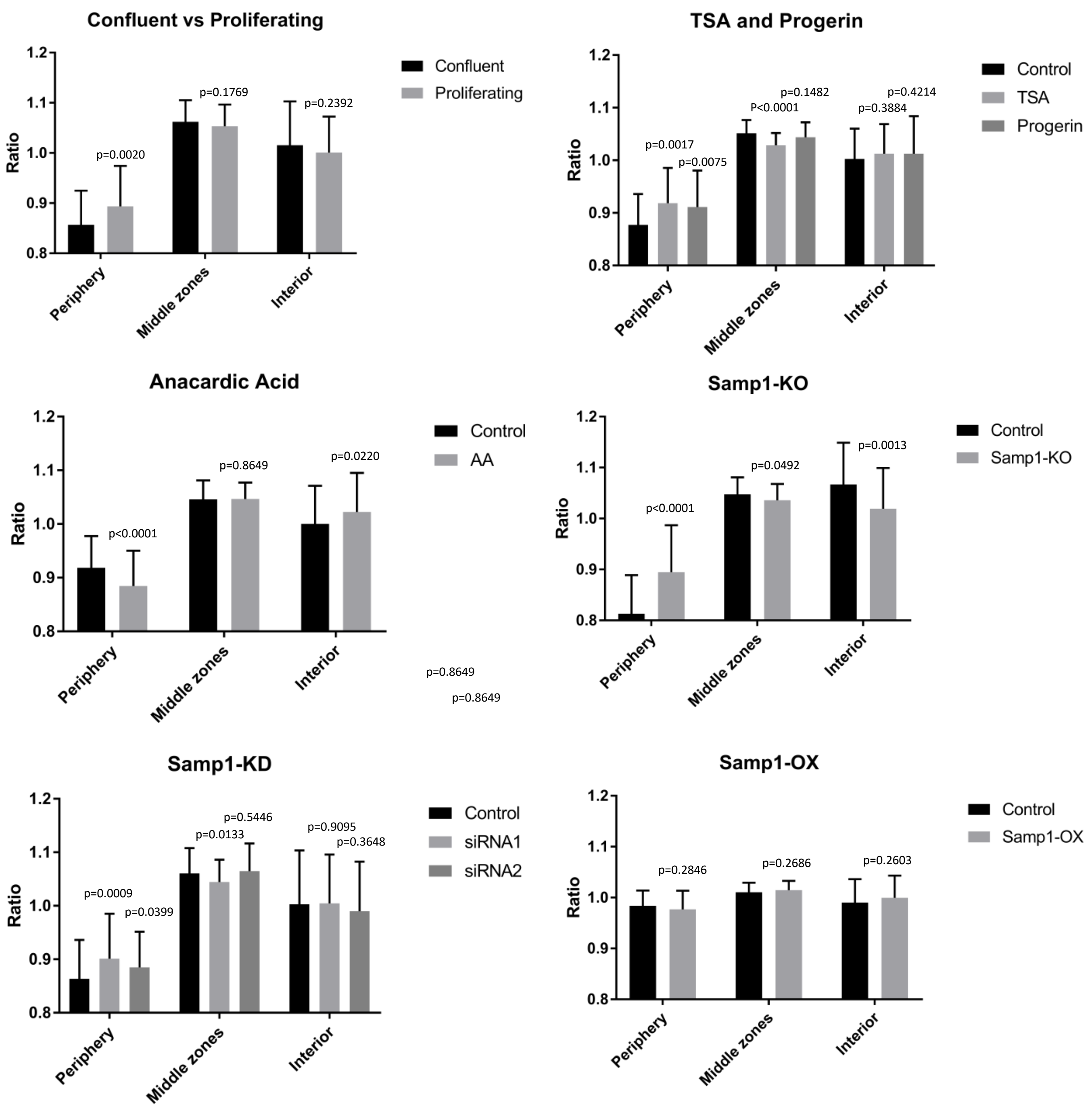

Supplementary Figure S2. Representation of data when ratio is measured in 3 zones of equal area. All nuclei were divided into 3 zones of equal area, and the mean signal intensities are given for each of the 3 zones. More subtle changes to chromatin organization in the nuclear periphery are lost representing the data in this way compared to in a radial profile with 40 zones of equal width, as can be seen for Samp1-OX cells.

Supplementary Figure S3

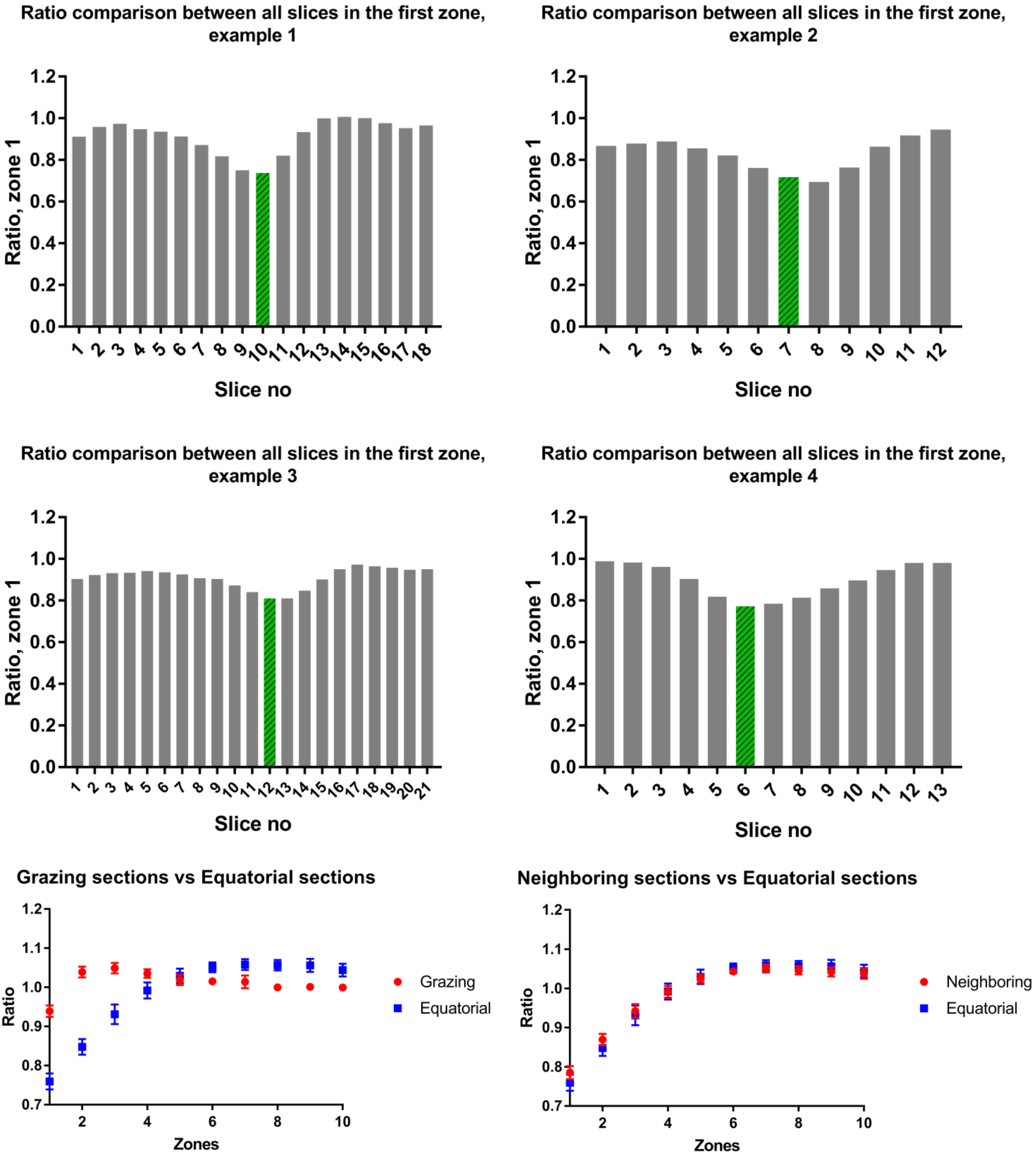

Supplementary Figure S3. Demonstration of which slices were chosen for analysis. Top 4 bar graphs illustrate the ratio values of the first zone in all slices of 4 individual cells. Green striped bar shows which slice had the largest surface area, and was the slice chosen for analysis (equatorial section). Radial profiles illustrate the ratio in the first 10 zones when comparing either the 2 outermost (grazing) slices to the equatorial slice, or the two neighboring slices to the equatorial slice. Error bars represent SEM, n=4 (equatorial slices) and n=8 (grazing or neighboring slices). Slice thickness= 0,5 µm for all cells. Different slice numbers occur due to variance in nuclear size.

Supplementary Figure S4

A

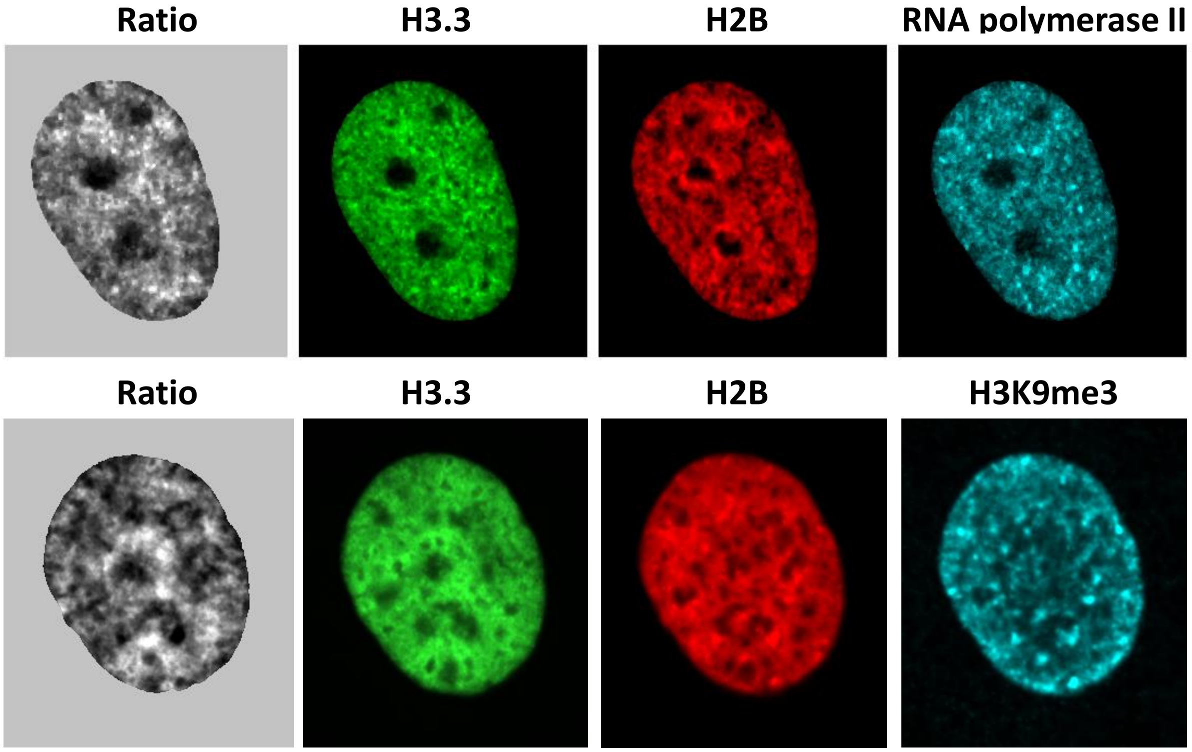

B

**Correlation with RNA polymerase II**	**Ratio (H3.3/H2B)**	**Inverse Ratio (H2B/H3.3)**	**H3.3**	**H2B**
Pearson Correlation Coefficient	0,302	-0,321	0,281	-0,054
Mander Costes Correlation Coefficient	0,711	0,373	0,654	0,043
**Correlation with H3K9me3**	**Ratio (H3.3/H2B)**	**Inverse Ratio (H2B/H3.3)**	**H3.3**	**H2B**
Pearson Correlation Coefficient	-0,320	0,271	-0,013	0,513
Mander Costes Correlation Coefficient	0,021	0,516	0,1168	0,786

Supplementary Figure S4. Distribution of H3.3/H2B ratio versus RNA polymerase II. U2OS cells transfected with pTandemH were fixed and immunostained for RNA polymerase II or heterochromatin marker H3K9me3. RNA polymerase II (RNApol II) and H3K9me3 were compared to the distribution of the H3.3/H2B ratio, H3.3-EGFP and H2B-mCherry alone. The ratio (H3.3/H2B) had a higher Pearson Correlation Coefficient (PCC) and Mander Correlation Coefficient (MCC) at a threshold of 75%, with RNA polymerase II than either H3.3-EGFP or H2B-mCherry alone, and a very low correlation was found between the inverse ratio (H2B/H3.3) and RNA Polymerase II. However, the H3K9me3 marker had a higher PCC and MCC with the inverse ratio and H2B-mCherry than either the ratio or H3.3-EGFP, at a threshold of 30%.

Supplementary Figure S5

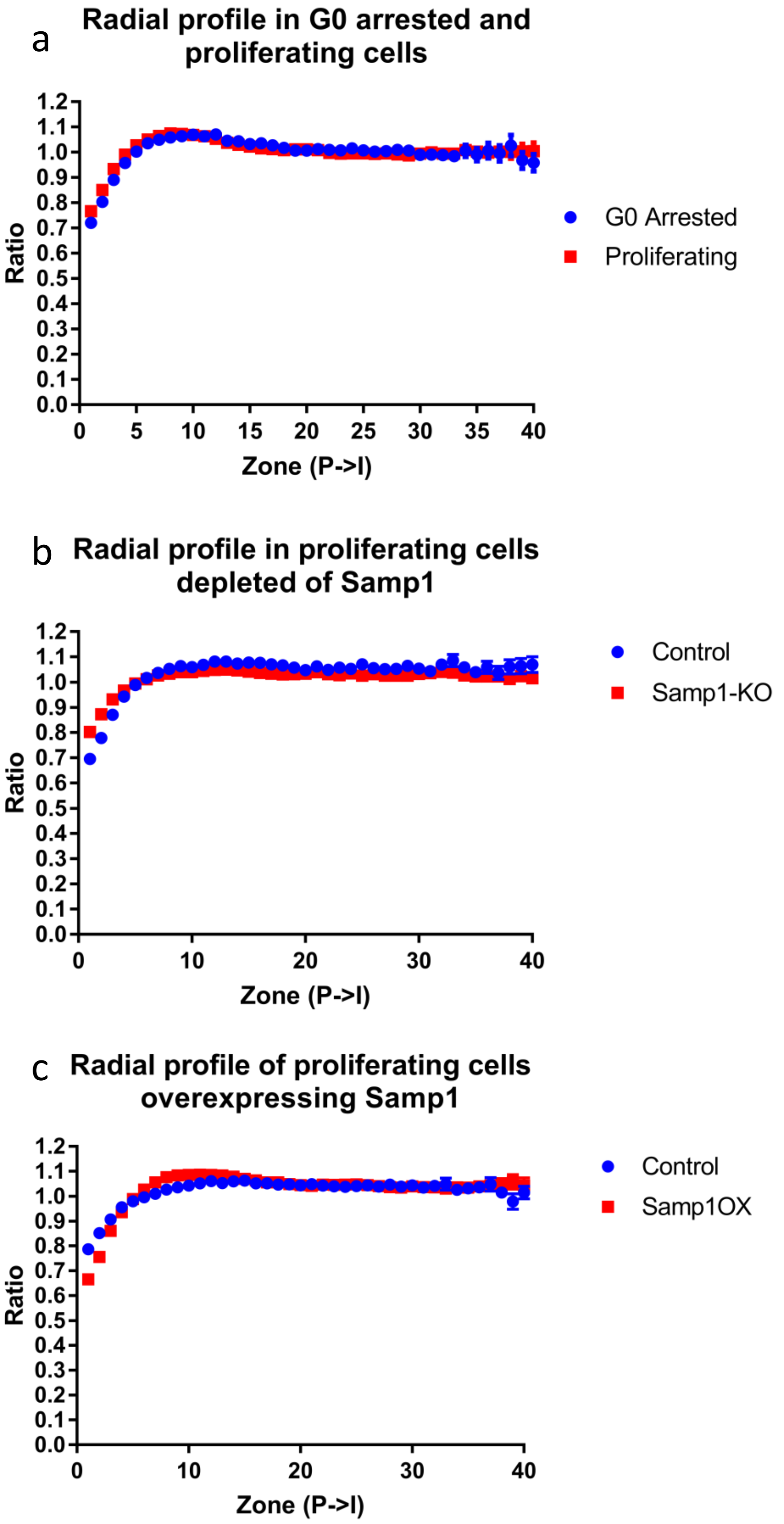

Supplementary Figure S5. Chromatin distribution in proliferating and G_0 Arrested U2OS cells. a) Radial profile (P→I; all zones) from experiment described in Fig. 2B. b) Radial profile (P → I; all zones) of proliferating U2OS cells depleted of Samp1. Zones 1-3 were statistically significant (p<0.01, n=109 controls, n=114 Samp1-KO cells) . c) Radial profile (P → I; all zones) of proliferating U2OS cells overexpressing Samp1. Zones 1-3 were statistically significant (p<0.0133, n=75 controls, n=62 Samp1OX cells). Experiments were performed three times.

Supplementary Figure S6

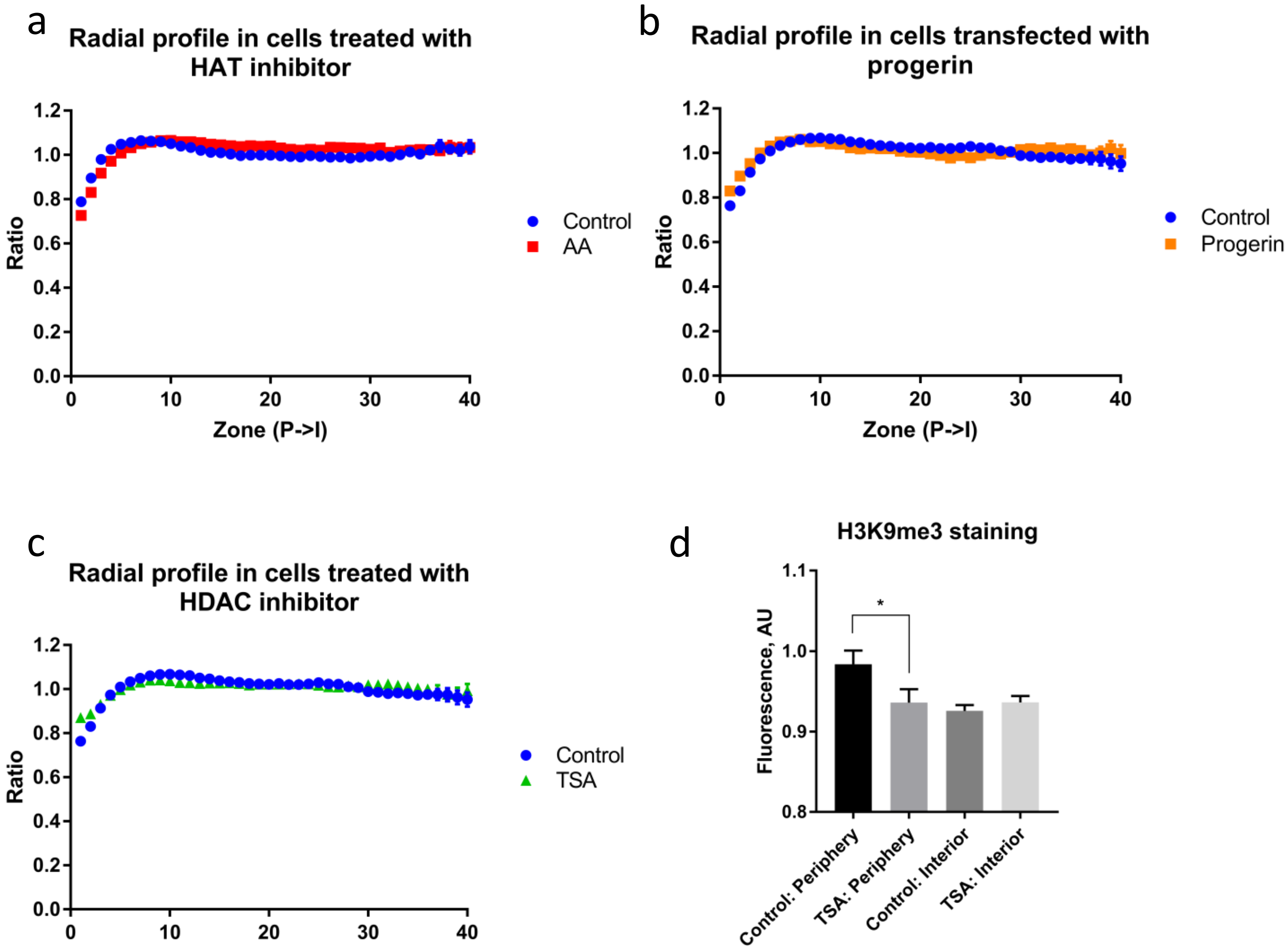

Supplementary Figure S6. Effects of histone acetylation and Progerin expression on chromatin organization in U2OS cells. Radial profile (P→I; all zones) from experiment described in Fig. 3B (a) Fig. 3H (b) and Fig. 3E (c). The bar graph represents data where an antibody against heterochromatin was used to confirm the ratio results concerning effects of TSA on chromatin distribution (p=0.0492, n=47 control cells, n=45 TSA cells) (d). Experiments performed three times.

Supplementary Figure S7

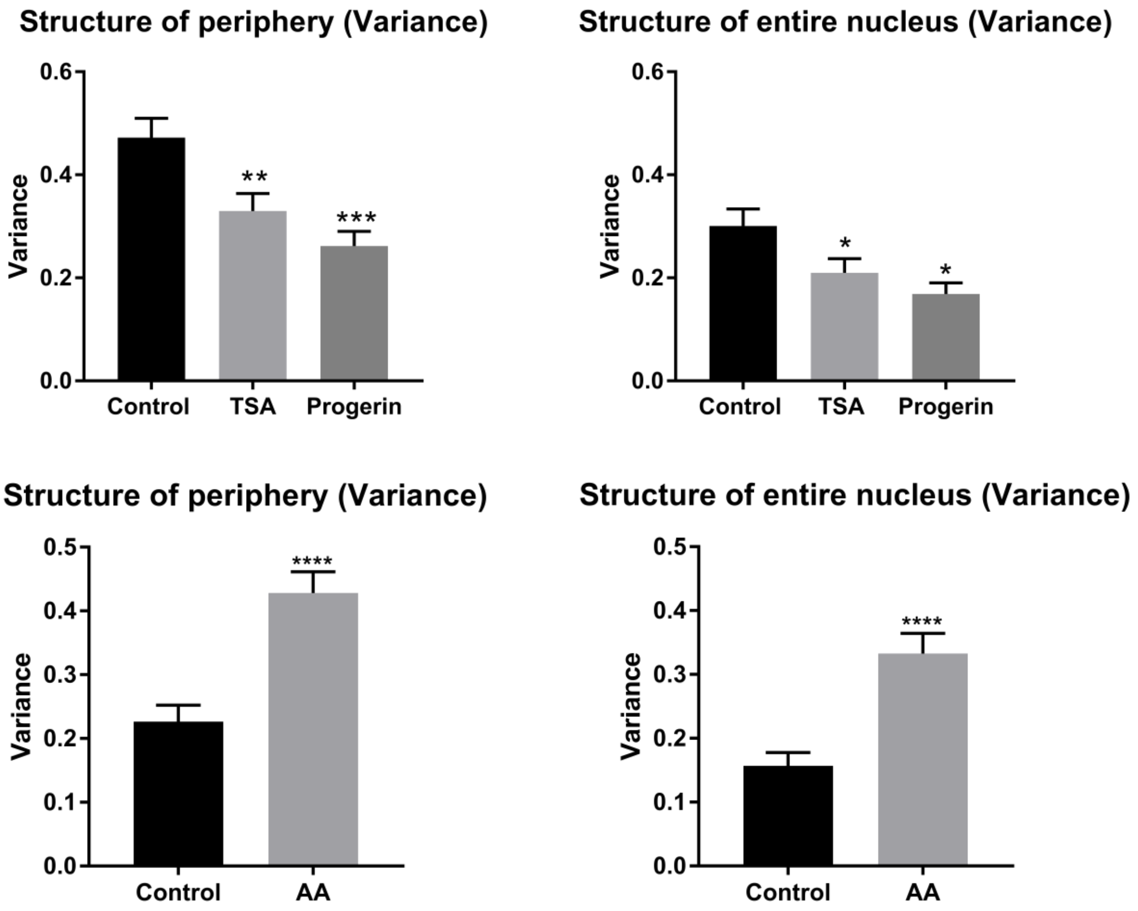

Supplementary Figure S7. Image structure of cells used for experiments applying TSA, progerin or Anacardic acid. Image structure of nuclear periphery (10 most peripheral zones) and the entire nuclei measured as variance of pixels in confluent U2OS cells treated with TSA or Anacardic acid, or cells transfected with R-plasmid encoding Progerin, compared to control cells.

Supplementary Figure S8

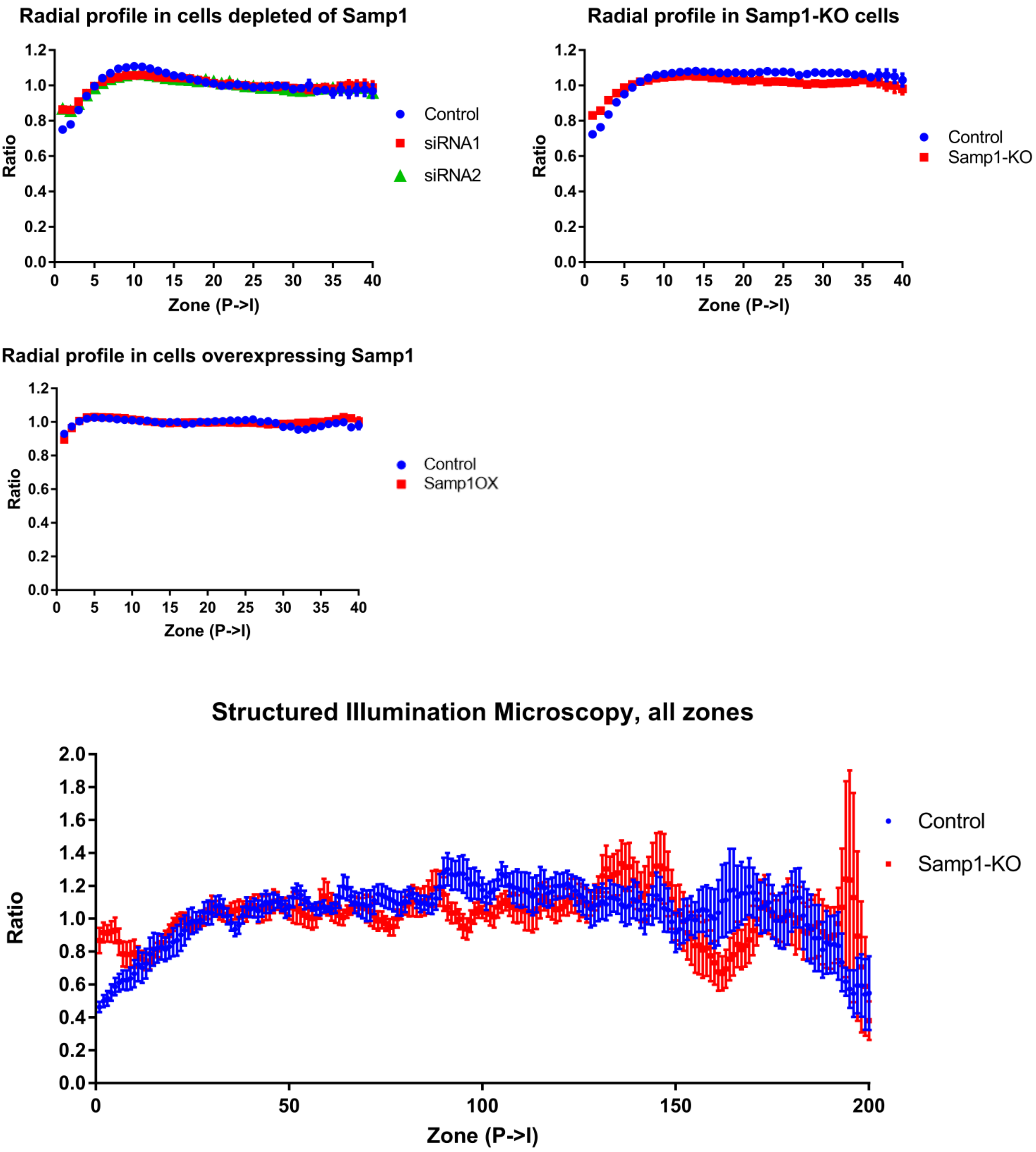

Supplementary Figure S8. Effects of Samp1 expression on chromatin organization in U2OS cells. Radial profile (P→I; all zones) from experiment described in Fig. 5B (upper left) Fig. 5F (upper right), Fig. 6A (middle) and Fig. 7B (lower). Results presented with SEM error bars.

Supplementary Figure S9

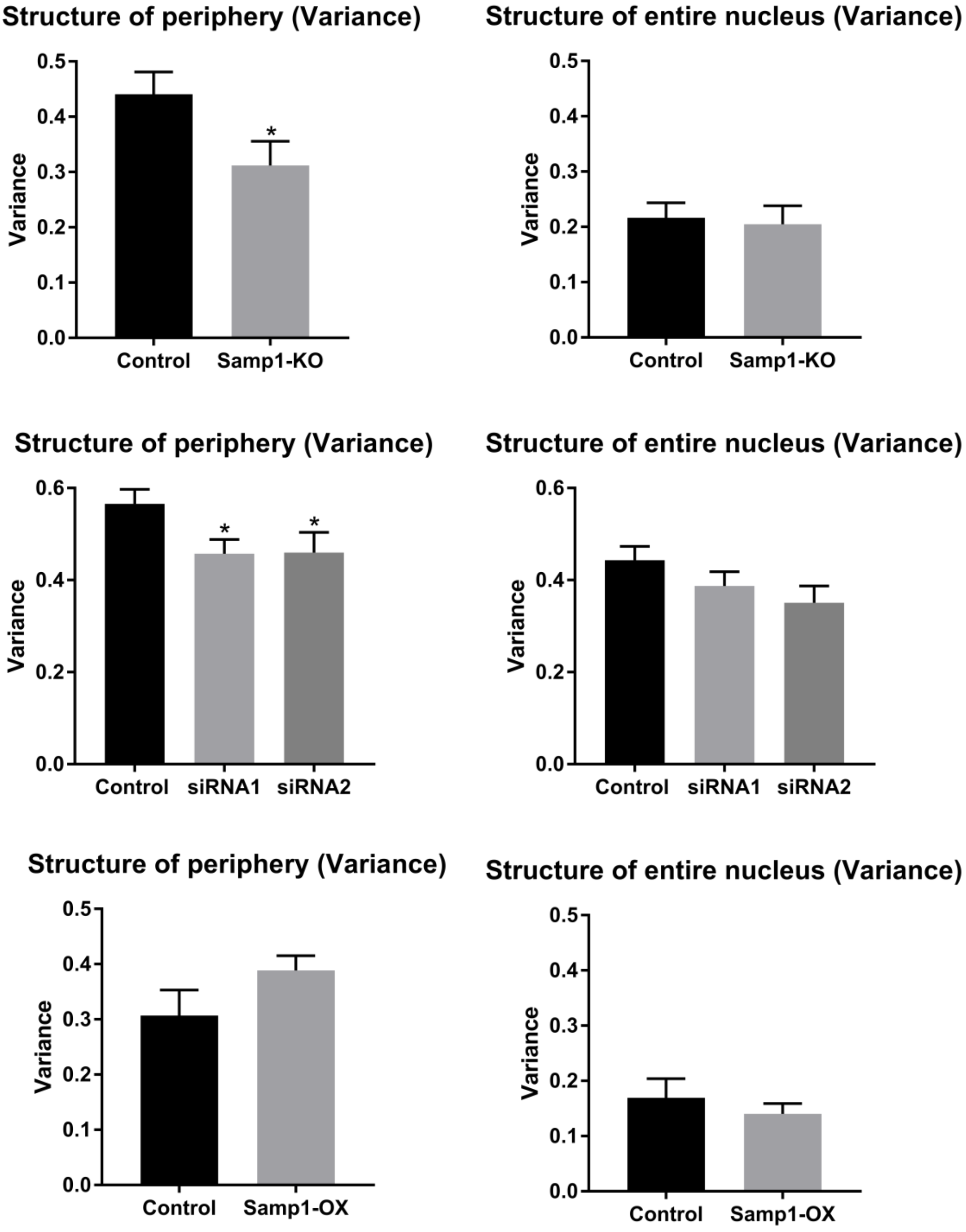

Supplementary Figure S9. Image structure of U2OS cells used for experiments investigating the effects of changes in Samp1 levels. Image structure of nuclear periphery (10 most peripheral zones) and the entire nuclei measured as variance of pixels in confluent cells depleted of Samp1 using siRNA or stable knock-out cell lines, or overexpressing Samp1, compared to scrambled control cells.
